# Resveratrol inhibits release of soluble fms-like tyrosine kinase (sFlt-1) and soluble endoglin and improves vascular dysfunction – implications as a preeclampsia treatment

**DOI:** 10.1038/s41598-017-01993-w

**Published:** 2017-05-12

**Authors:** Natalie J. Hannan, Fiona C. Brownfoot, Ping Cannon, Minh Deo, Sally Beard, Tuong V. Nguyen, Kirsten R. Palmer, Stephen Tong, Tu’uhevaha J. Kaitu’u-Lino

**Affiliations:** 1 Translational Obstetrics Group, The Department of Obstetrics and Gynaecology, Mercy Hospital for Women, University of Melbourne, 163 Studley Rd, Heidelberg, 3084 Victoria Australia; 2 Department of Obstetrics and Gynaecology, Monash University, Monash Medical Centre, 246 Clayton Rd, Clayton, 3168 Victoria Australia

## Abstract

Preeclampsia is a disease of pregnancy associated with placental oxidative stress, inflammation and elevated release of anti-angiogenic factors sFlt-1 and soluble endoglin. These placental factors cause generalized maternal endothelial dysfunction. There are no treatments to halt disease progression; delivery is the only cure. Resveratrol modulates pathways involved in inflammation and oxidative stress and may offer a potential therapeutic for preeclampsia. Resveratrol reduced sFlt-1, sFlt-1 e15a and soluble endoglin secretion from primary trophoblasts and HUVECs and reduced mRNA expression of pro-inflammatory molecules NFκB, IL-6 and IL-1β in trophoblasts. IL-6, IL-1β and TNFα secretion were also significantly reduced. In HUVECs, resveratrol significantly increased mRNA of anti-oxidant enzymes HO-1, NQO1, GCLC and TXN but did not significantly alter HO-1 protein expression, whilst reducing HO-1 protein in trophoblast. Endothelial dysfunction was induced in HUVECs using TNFα, increasing expression of cell adhesion molecule VCAM1 and adhesion of peripheral blood mononuclear cells, both of which were increased further by resveratrol. In contrast, resveratrol significantly reduced TNFα-induced Endothelin-1 (a vasoconstrictor) and significantly increased the phosphorylation of endothelial nitric oxide synthase (eNOS). In summary, resveratrol decreases secretion of anti-angiogenic factors however its effects on the endothelium are mixed. Overall, it may have potential as a treatment for preeclampsia.

## Introduction

Preeclampsia is a pregnancy complication, globally responsible for tens of thousands of maternal and neonatal deaths each year^1, 2^. Important steps in the pathogenesis of preeclampsia are poor placental invasion and the subsequent release of anti-angiogenic factors such as soluble fms-like tyrosine kinase 1 (sFlt-1)^3–5^ and soluble endoglin (sEng)^6^ into the maternal circulation. These anti-angiogenic factors cause widespread endothelial dysfunction leading to multisystem organ injury^7–9^. Currently, there are no medical therapies to halt disease progression and expectant management and delivery remain the mainstay of treatment^1^.

Resveratrol is a naturally occurring agent found in a number of fruits, with its most commonly reported source being the skin of red grapes. It is an anti-oxidant molecule that also has anti-inflammatory properties^10, 11^. Throughout the last decade, resveratrol has been assessed for its potential therapeutic effects in various diseases including cancer, autoimmune and degenerative diseases^12^.

The effects of resveratrol in mouse models relevant to preeclampsia have been reported. Poudel *et al*.^13^ assessed resveratrol in two mouse models; the endothelial nitric oxide synthase (eNOS) and Catechol-O-Methyltransferase (COMT) knockout mice^13, 14^. While they did not see a change in blood pressure in either model (the mice were not hypertensive to start with), resveratrol prevented the occurrence of proteinuria characteristic of the eNOS mouse and clinical preeclampsia. In regards to fetal size, resveratrol did not rescue the reduced fetal size of the eNOS pups, however increased fetal weight and crown-rump length in the COMT model. Neither model showed any increases in placental weight or size following resveratrol treatment. With the addition of vasoconstrictor methacholine in eNOS mice, uterine artery relaxation was impaired and this was improved with the addition of resveratrol^13^. Resveratrol has also been reported to reduce high blood pressure in the L-N^G^-Nitroarginine methyl ester (L-NAME) rat model of preeclampsia and reverse key markers of oxidative stress, however it did not reduce proteinuria or change fetal weight or placental weights in this model^15^.

Furthermore pre-clinical data supports the concept that resveratrol may be a treatment for preeclampsia^16^. Reduced sFlt-1 secretion from placental explants was observed when treated with resveratrol, in addition this was also seen in the presence of TNFα and VEGF. When resveratrol was administered to HTR8s (placental cell line) in the presence of angiotensin II, TNFα or phorbol-12-myristate-13-acetate (PMA) (a NFκB activator) there was a significant reduction in sFlt-1 secretion although this was not achieved when resveratrol was administered alone. Resveratrol also reduced sFlt-1 secretion from endothelial cells alone, and in the presence of VEGF, Interferon (IFN)-γ and PMA. In endothelial cells it was found to up-regulate heme-oxygenase 1 (HO-1)^16^.

Although this previous study^16^ suggests positive effects on sFlt-1 secretion, there is no information regarding the effect on sFlt-1 splice variant expression and those studies were only performed in placental cell lines (which we have shown secretes very little amounts of sFlt-1)^17^. Moreover, the effect of resveratrol on the other anti-angiogenic factor of preeclampsia, sEng, has not been reported. In this study we sought to assess the effects of resveratrol on both sFlt-1 and sEng secretion from primary isolated trophoblast and primary endothelial cells. In addition, we assessed whether resveratrol exerted anti-inflammatory and anti-oxidative effects in primary trophoblast as well as assessing whether it rescued TNFα – induced endothelial dysfunction, or upregulated the expression of phosphorylated endothelial nitric oxide synthase (eNOS, the active form that produces the vasodilator NO).

## Methods

### Isolation and treatment of primary human cytotrophoblasts

This study was approved by The Mercy Health Human Research Ethics Committee and all women gave written informed consent for the collection of samples. All methods were performed in accordance with the University of Melbourne and Mercy Health guidelines and regulations. Human cytotrophoblasts were isolated from term caesarean section placentas as previously described^18^. Primary cytotrophoblasts were cultured in DMEM high Glutamax (Life Technologies) containing 10% FCS and 1% antibiotic-antimycotic (Life Technologies) on fibronectin (10 mg/mL; BD Biosciences, New South Wales, Victoria) coated wells. Cells were plated and allowed to attach over 12–18 h before washing with dPBS (Life Technologies) to remove cell debris. Cells were cultured under 8% O_2_, 5% CO_2_ at 37 °C for 48 h following treatment.

### Isolation and treatment of primary human umbilical vein endothelial cells (HUVECs)

Umbilical cords were collected from term normal placenta and the cord vein was infused with 10 ml (1 mg/ml) of collagenase (Worthington, Lakewood, New Jersey) and cells isolated as previously described^19^. Cells were used between passage 2 to 4 and cultured at 37 °C in 20% O_2_ and 5% CO_2_.

### Resveratrol treatment

Resveratrol (Sigma, St Louis, Missouri, USA) was dissolved in ethanol according to manufacturer’s instructions and all control and treatment wells received equal volumes of ethanol to ensure that any effects of the vehicle were controlled for (final concentration of 0.04% ethanol). Primary trophoblast were treated with 0–100 μM Resveratrol (Sigma) for 48 h in culture. At the cessation of treatment, cell viability was assessed using a MTS assay (Promega, Madison, WI), media was collected for ELISA analysis and RNA or protein collected for down-stream analyses.

Primary HUVECs for measurement of sFlt-1 and sEng, or protein expression of peNOS, eNOS or HO-1 were treated with 0–75 μM Resveratrol for 24 h in culture. At the cessation of treatment, cell viability was assessed using a MTS assay, media was collected for ELISA analysis and RNA or protein collected for down-stream analyses.

### Cell viability assay (MTS assay)

Cell viability assays were performed using CellTiter 96-Aquesous One solution (Promega, Madison WI) according to the manufacturer’s instructions.

### ELISA Analysis

Concentrations of sFlt-1 and sEng were measured in conditioned cell/tissue culture media using the DuoSet Human VEGF R1/Flt-1, or DuoSet Human Endoglin kit (R&D systems by Bioscience, Waterloo, Australia) as per manufacturer’s instructions.

For ET-1 analysis, the Human Endothelin-1 Quantikine ELISA kit (R&D Systems) was used according to manufacturer’s instructions.

### Luminex Analysis

The cytokines, IL-1β, IL-6 and TNF-α were measured using quantitative Milliplex Luminex (MilliPlex MAP Human Cytokine Panel, Millipore, Melbourne, Victoria, Australia) assays according to the manufacturer’s instructions. 96-well Milliplex plates were pre-wet with 200 μl assay buffer (provided by the manufacturer) for 10 minutes and then aspirated using a vacuum manifold. Standards and samples (25 μl) were added to appropriate wells, followed by the addition of assay beads. Plates were incubated overnight for 16–18 h with mild agitation at 4 °C; the fluid was then removed by vacuum and the wells were washed twice with wash buffer. Detection antibodies were added to each well, and incubated for 1 h at room temperature (RT), the fluorescent conjugate Streptavidin-Phycoerythrin was added to each well and plates incubated for 30 min at RT. Fluid was then removed by vacuum and wells washed twice. Analysis of each sample was performed in duplicate. Identical positive and negative quality controls are included on each assay in duplicate.

### Endothelial dysfunction

Endothelial dysfunction experiments were undertaken using primary HUVECs. HUVECs were pre-treated with 1 ng/ml TNFα (Sigma) for 2 h, before increasing doses of resveratrol (0–75 μM) was added in the presence of TNFα for a further 24 h. At the cessation of the experiment, media was collected for ELISA analysis of endothelin-1 (ET-1) and RNA collected for qRT-PCR measurement of VCAM and ET-1.

#### Adhesion Assay

HUVECs were treated with a constant dose of 1 ng/ml TNFα (Sigma) with increasing doses of resveratrol (0–75 μM) for 24 hours at 20% O_2_, 5% CO_2_ and 37 °C. For the leukocyte adhesion assay experiment, primary human Peripheral Bone Marrow Cells (PBMCs) were isolated from the whole blood of pregnant patients. Blood was collected in an EDTA vacutainer and centrifuged to remove plasma. Red blood cell fraction was diluted with PBS and layered on top of 12 ml Ficoll-Paque (GE Healthcare, Little Chalfont, UK). After centrifugation at 400 g for 30 mins without brakes, the PBMC fraction was collected and washed in PBS to remove excess Ficoll-Paque. Contaminating platelets were removed following a second centrifugation at 300 × g for 10 mins. Red blood cells were lysed and PBMCs collected. PBMCs were pre-incubated with calcein (Merk Millipore, Darmstadt, Germany) for 30 minutes at 37 °C and applied to HUVECs as previously described^20^. Fluostar omega fluorescent plate reader (BMG labtech, Victoria, Australia) was used to detect fluorescence (quantify adhesion).

### Quantitative RT-PCR

RNA was extracted from primary trophoblast and HUVECs using an RNeasy mini kit (Qiagen, Valencia, CA) and quantified using the Nanodrop ND 1000 spectrophotometer (NanoDrop technologies Inc, Wilmington, DE). 0.2 μg of RNA was converted to cDNA using Applied Biosystems high capacity cDNA reverse transcriptase kit (Life Technologies) as per manufacturer guidelines.

Gene expression of *VCAM-1, ET-1, MMP 14, HO-1, Endoglin, TIMP3, NQO1, GCLC, TXN, YWHAZ and GAPDH* (Life Technologies) were quantified by real time PCR (RT-PCR) on the CFX 384 (Bio-Rad, Hercules, CA) using FAM-labeled Taqman universal PCR mastermix and its specific primer/probe set (Life Technologies) with the following run conditions: 50 °C for 2 minutes; 95 °C for 10 minutes, 95 °C for 15 seconds, 60 °C for 1 minute (40 cycles). SYBR RT-PCR was carried out to assess gene expressions of *sFlt-1 e15a* and *sFlt-1 i13*, *YWHAZ* and *GAPDH*. Primers were designed as previously described (Geneworks, South Australia, Australia)^21^. RT-PCR was performed using the following run conditions: 95 °C for 20 minutes; 95 °C for 0.01 minutes, 60 °C for 20 minutes, 95 °C for 1 minute (39 cycles), melt curve 65 °C to 95 °C at 0.05 °C increments at 0.05 seconds.

### HO-1 and p-eNOS/eNOS Western Blot

20 µg of placental lysates were separated on 10% polyacrylamide gels with wet transfer to PVDF membranes (Millipore, Billerica, MA). Membranes were blocked prior to them being divided in to 3 sections and being incubated overnight with either ***anti-HO-1*** (ADI-SPA-896; ENZO Life Sciences: 1:500 dilution), ***anti-p-eNOS*** (pT495, phospho-specific (612706); Becton Dickinson Biosciences, Franklin Lakes, NJ: 1:200 dilution), or ***actin*** (Santa Cruz, SC, USA 1:2000 dilution). Bands were visualized using a chemiluminescence detection system (GE Healthcare Life Sciences) and ChemiDoc XRS (BioRad, Hercules, CA). Following imaging, the anti-p-eNOS blot was stripped with stripping buffer and probed overnight with ***anti-eNOS*** (eNOS/NOS Type III (610298); Becton Dickinson Biosciences: 1:500 dilution). GAPDH was used as a loading control. Relative densitometry was determined using QuantityOne software (BioRad).

### Statistical analysis

Triplicate technical replicates were performed for each experiment, with a minimum of three independent biological replicates performed for each *in vitro* study. A power calculation was not undertaken. Data was tested for normal distribution and statistically analyzed as appropriate. When three or more groups were compared a 1-way ANOVA (for parametric data) or Kruskal-Wallis test (for non-parametric data) was used. Post-hoc analysis was carried out using either the Tukey (parametric) or Dunn’s test (non-parametric). When two groups were analyzed, either an unpaired t-test (parametric) or a Mann Whitney test (non-parametric) was used. All data is expressed as mean ± SEM. P values < 0.05 were considered significant. Statistical analysis was performed using GraphPad Prism 7 software (GraphPad Software, La Jolla, CA).

## Results

### Resveratrol reduces sFlt-1 and sEng secretion from primary cytotrophoblasts

It is thought that elevated secretion of sFlt-1 and sEng into the maternal circulation is responsible for the significant maternal endothelial dysfunction that occurs in preeclampsia^3, 4, 6^. Here, we assessed whether resveratrol can reduce sFlt-1 and/or sEng secretion from primary cytotrophoblasts.

**Figure 1:**
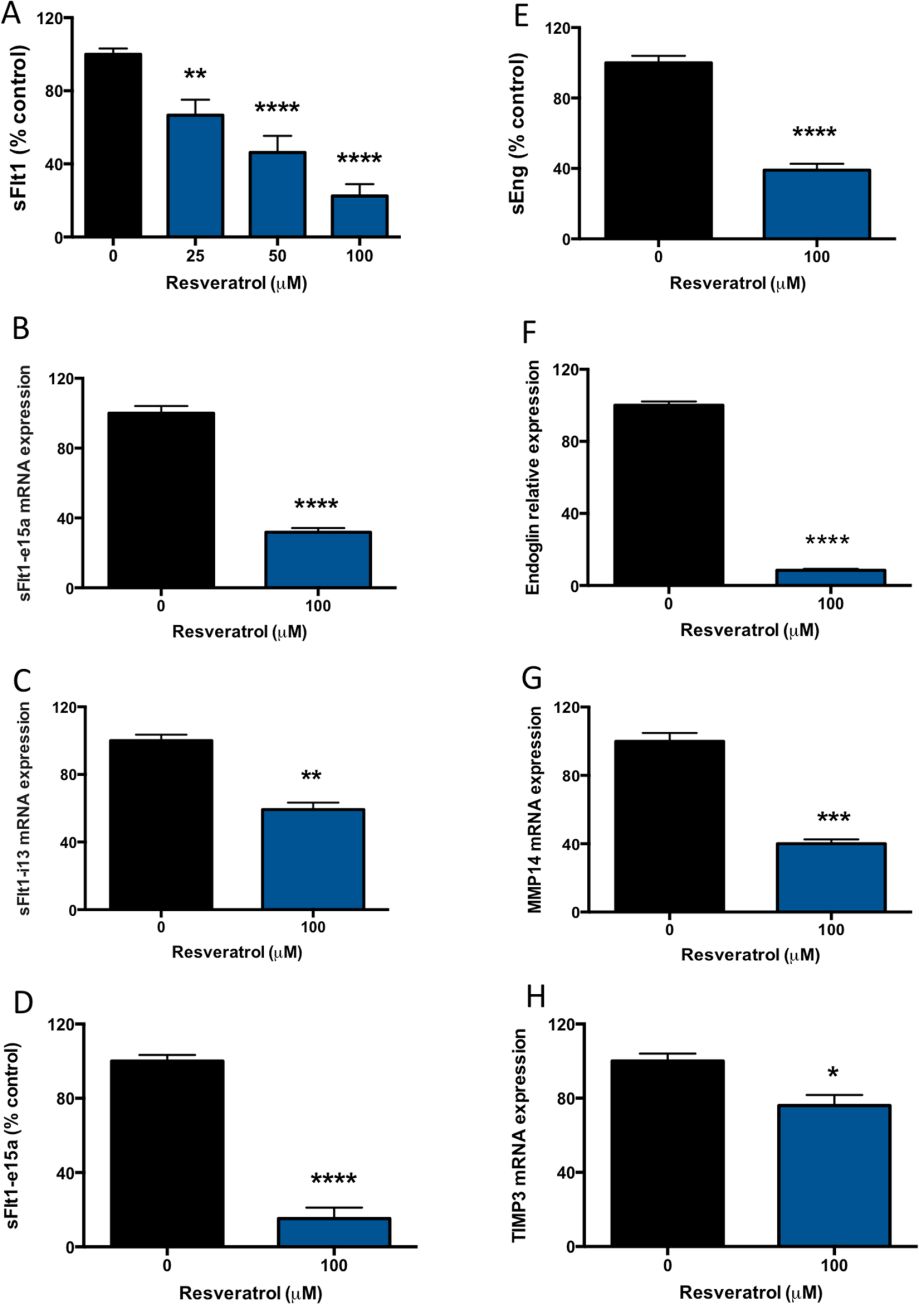
Resveratrol significantly reduces sFlt-1, sFlt-1 e15a and sEng secretion from primary cytotrophoblast. Resveratrol was added at increasing doses to primary trophoblast and the effect on sFlt-1 and sEng secretion determined. (**A**) Resveratrol induced a significant reduction in sFlt-1 secretion at 25–100 μM. This was accompanied by a (**B**) significant reduction in the sFlt-1 placental variant, sFlt-1 e15a mRNA expression and a (**C**) significant reduction in sFlt-1 i13 mRNA expression. Given the marked effect on sFlt-1 e15a mRNA expression, we subsequently measured secretion of sFlt-1 e15a using our in-house ELISA and found that resveratrol potently reduced sFlt-1 e15a secretion (**D**). sEng is the other important anti-angiogenic factor associated with preeclampsia. (**E**) Resveratrol significantly reduced sEng secretion from primary trophoblast. This was accompanied by significantly reduced endoglin (**F**), MMP14 (**G**) and TIMP3 (**H**) mRNA expression. Data expressed as mean ± SEM. *p < 0.05 **p < 0.01, ***p < 0.001, ****p < 0.0001 compared to 0 resveratrol. N = 3–5 experimental replicates, with each n taken from a separate patient isolation.

In primary cytotrophoblast, resveratrol induced a significant dose-dependent reduction in sFlt-1 secretion, quenching secretion by approximately 80% at the top dose of 100 μM (Fig. 1A) without negatively impacting cell viability (Supplementary Figure 1A). In placenta, more than 90% of sFlt-1 transcripts comprises the splice variant sFlt-1 e15a, whilst sFlt-1 i13, is expressed to a lesser extent^22^. Here we show that resveratrol also significantly reduces the mRNA expression of both sFlt-1 e15a (Fig. 1B) and sFlt-1 i13 (Fig. 1C) in primary cytotrophoblasts. Given the marked effect on sFlt-1 e15a mRNA expression, we also measured secretion of sFlt-1 e15a using our in-house generated ELISA we have previously described^23^. We found resveratrol potently reduced sFlt-1 e15a secretion (by approximately 85%) from primary cytotrophoblast (Fig. 1D).

To our knowledge, the effect of resveratrol on sEng secretion has not been previously assessed. Resveratrol induced a 60% reduction in sEng secretion from primary cytotrophoblast (Fig. 1E). We previously reported that membrane bound endoglin is cleaved at the placental surface by matrix metalloproteinase 14 (MMP14; a membrane bound protease) to release sEng^17^. As such, we measured the mRNA expression of total endoglin, MMP14 and its natural inhibitor Tissue Inhibitor of Metalloproteinase 3 (TIMP3) following resveratrol treatment. We found resveratrol induced a potent reduction in total endoglin (Fig. 1F), whilst also reducing MMP14 (Fig. 1G) and TIMP3 mRNA expression (Fig. 1H).

We have found resveratrol significantly reduced both sFlt-1 and sEng secretion from primary trophoblast, possibly via its ability to reduce the expression of sFlt-1 splice variants, reduce total endoglin and expression of its cleavage protease, MMP14.

### Resveratrol also reduces sFlt-1 and sEng secretion from primary HUVECs

**Figure 2:**
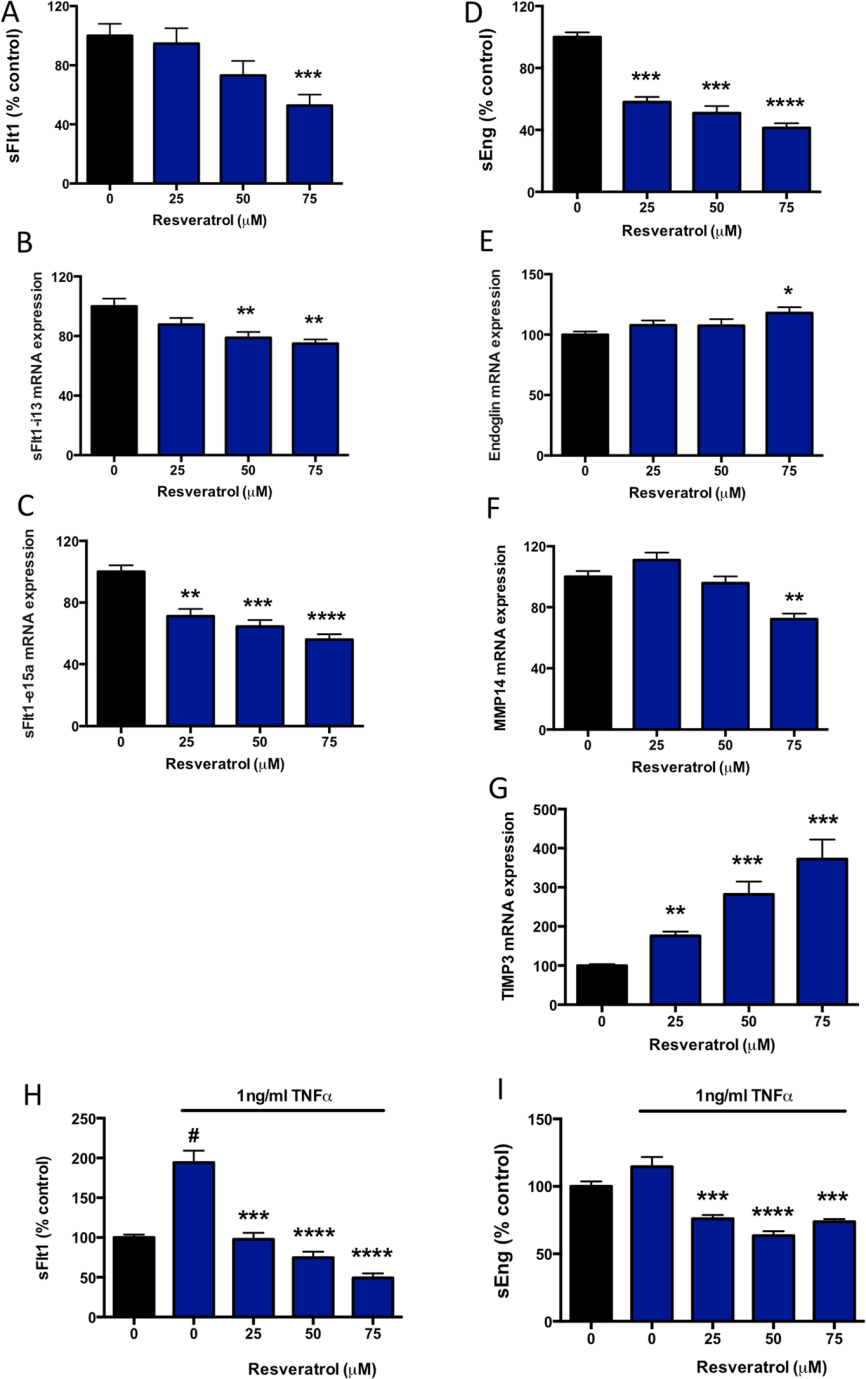
Resveratrol significantly reduces sFlt1 and sEng secretion from primary HUVECs. Resveratrol was added at increasing doses to primary HUVECs under basal conditions (**A–G**) or after addition of TNFα (H,I), and the effect on sFlt-1 and sEng secretion was determined. (**A**) Resveratrol induced a significant reduction in sFlt-1 secretion at 75 μM. This was accompanied by a (**B**) significant reduction in sFlt-1 ei13 mRNA expression and a (**C**) significant reduction in sFlt-1 e15a mRNA expression. We also found that resveratrol significantly reduced sEng secretion from primary HUVECs (**D**). This was accompanied by significantly increased endoglin (**E**), mRNA expression. (**F**) In primary HUVECs, resveratrol significantly reduced MMP14 expression whilst (**G**) upregulating expression of its natural inhibitor TIMP3. We also assessed the effect of resveratrol in the presence of TNFα (**H,I**). TNFα significantly increased sFlt-1 secretion, and this effect was reversed by treatment with resveratrol (**H**). In contrast TNFα did not significantly alter sEng secretion, however resveratrol still significantly reduced secretion in the presence of TNFα (**I**). Data expressed as mean ± SEM. For **A–G**, *p < 0.05 **p < 0.01, ***p < 0.001, ****p < 0.0001. For **H,I**, ^#^p < 0.05 compared to control, ***p < 0.001, ****p < 0.0001 compared to TNFα alone. N = 3–5 experimental replicates, with each n taken from a separate patient isolation.

Previous data^16^ demonstrates that resveratrol induces a significant reduction in sFlt-1 protein secretion from primary HUVECs. We demonstrate that resveratrol dose dependently reduced sFlt-1 secretion (Fig. 2A) in a dose-range of 25–75 μM. Of note, 100 μM resveratrol significantly reduced viability of primary HUVECs in our hands (Supplementary Figure 1B). We also found that resveratrol significantly reduced sFlt-1 i13 (Fig. 2B) and sFlt-1 e15a (Fig. 2C) mRNA expression. Of interest, in primary HUVECs we found that GAPDH mRNA expression was significantly regulated in a dose-dependent manner by resveratrol treatment. We therefore used an alternative housekeeping gene, YWHAZ, to perform our PCR analyses in HUVECs.

Given resveratrol significantly reduced sEng secretion from primary cytotrophoblast we next examined whether this is the case in endothelial cells, (an abundant source of sEng secretion). Indeed, resveratrol induced a significant dose dependent reduction in sEng secretion from primary HUVECs (Fig. 2D). In contrast to primary cytotrophoblasts, resveratrol stimulated endoglin mRNA expression at 75 μM (Fig. 2E), significantly reduced MMP14 expression at 75 μM (Fig. 2F) and induced a potent dose dependent increase in the MMP14 inhibitor, TIMP3 (Fig. 2G).

We also measured the effect of resveratrol on secretion of sFlt-1 and sEng from primary HUVECs in the presence of TNFα (Fig. 2H,I). MTS assay initially demonstrated a minor but significant reduction in the presence of TNFα compared to control. Importantly however, there were no significant differences in viability when comparing TNFα alone to the TNFα +/− resveratrol treated samples (Supplementary Figure 1C). 1 ng/ml TNFα significantly increased sFlt-1 secretion compared to control, and this effect was dose dependently reduced following resveratrol treatment. Although TNFα did not significantly increase sEng secretion, treatment with resveratrol significantly reduced sEng secretion from Primary HUVECs in the presence of TNFα.

Thus, we confirmed resveratrol reduced sFlt-1 secretion (as well as expression of the two main splice variants in endothelial cells)^22^ and sEng secretion from endothelial cells under both basal conditions and in the presence of TNFα.

### Effect of resveratrol on anti-oxidant molecules

Nuclear factor (erythroid-derived 2)-like 2 (Nrf2) is a transcription factor that binds to the anti-oxidant responsive element^24–26^. Nrf2 is sequestered in the cytoplasm through binding to the KEAP1 protein. When released, Nrf2 is phosphorylated, allowing its translocation to the nucleus causing an up-regulation in anti-oxidant genes (including HO-1, NAD(P)H dehydrogenase Quinone 1 (NQO1), Glutamate—cysteine ligase catalytic subunit (GCLC) and thioredoxin (TXN)). Previously it has been shown that resveratrol increases HO-1 expression in endothelial cells^16^.

**Figure 3:**
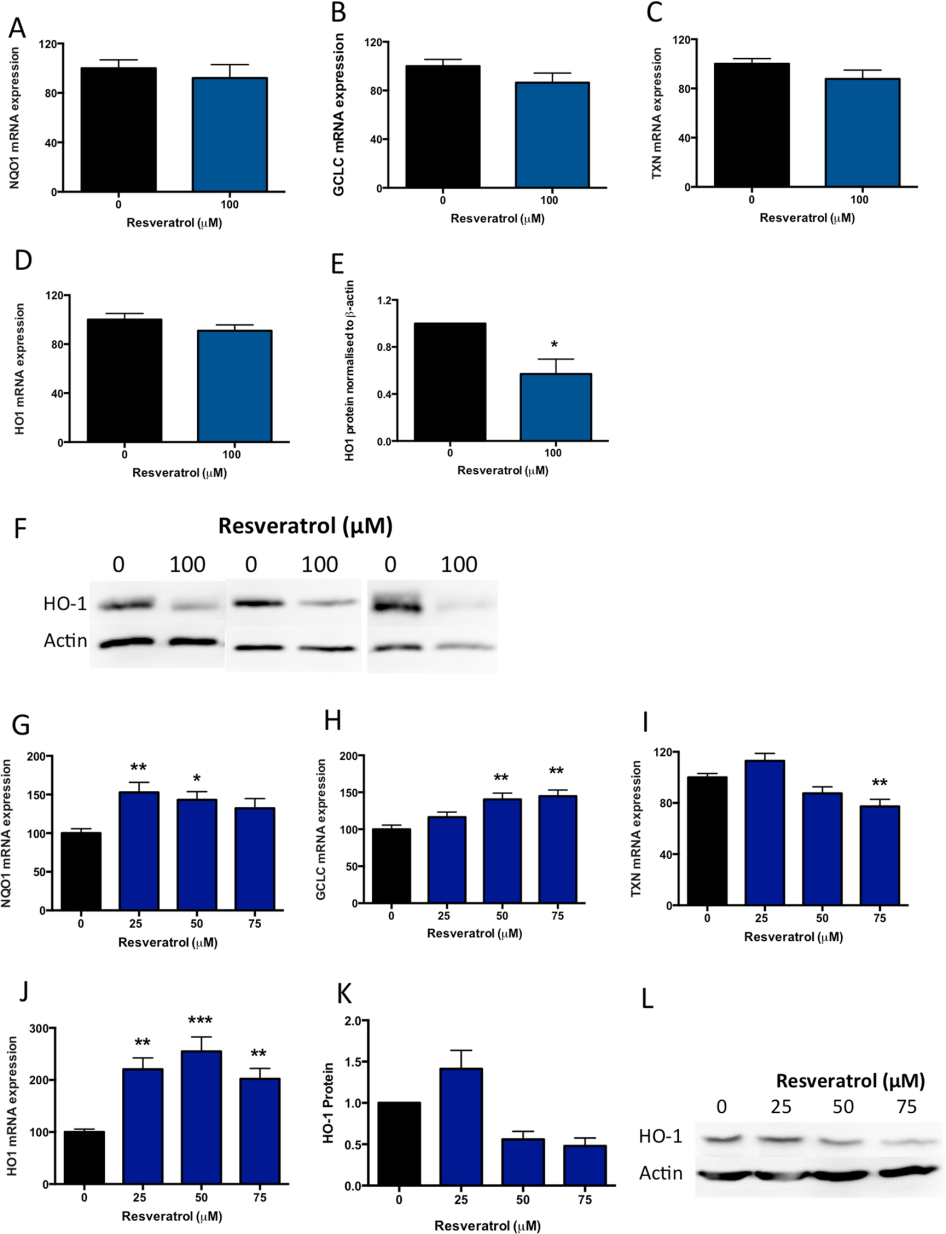
Resveratrol stimulates anti-oxidant molecules in primary HUVECs, but not primary cytotrophoblasts. In primary cytotrophoblast, resveratrol did not significantly alter anti-oxidant response element (ARE) genes (**A**) NQO1, (**B**) GCLC, (**C**) TXN, or (**D**) HO-1 mRNA expression, but significantly reduced HO-1 protein expression assessed via western blot with densitometric analysis (**E–F**). In primary HUVEC, resveratrol significantly induced mRNA expression of (**G**) NQO1, (**H**) GCLC, and (**J**) HO1, but significantly reduced (**I**) mRNA expression of TXN. (**K**,**L**) Resveratrol did not significantly alter HO-1 protein expression in primary HUVECs. Data expressed as mean ± SEM. *p < 0.05, **p < 0.01, ***p < 0.001 compared to 0 resveratrol (control). N = 3–5 experimental replicates, with each n taken from a separate patient isolation. F shows a western blot from 3 separate cytotrophoblast isolations, L shows a representative western blot from n = 1 HUVEC experiment, whilst K depicts densitometric analysis of n = 3 separate HUVEC western blots. As indicated in the materials and methods, western blots were cut before antibody exposure and therefore cropped blots are displayed.

Here we examined whether resveratrol induced anti-oxidant molecules in primary trophoblast. We first examined the expression of Nrf2 responsive genes NQO1 (Fig. 3A), GCLC (Fig. 3B) and TXN (Fig. 3C) and HO-1 (Fig. 3D), and found resveratrol did not significantly alter their expression. Surprisingly, western blot analysis however demonstrated a significant reduction in HO-1 protein expression in primary trophoblast (Fig. 3E,F).

Given this data, we examined effects of resveratrol on these same anti-oxidant genes in primary HUVECs. Whilst we found resveratrol significantly increased Nrf2 responsive genes, NQO1 (Fig. 3G), GCLC (Fig. 3H) and HO-1 (Fig. 3J), but reduced TXN expression (Fig. 3I). Resveratrol had no significant effect on HO-1 protein expression in primary HUVECs (Fig. 3K,L) and we confirmed cell viability via MTS (Supplementary Figure 1D).

We conclude that resveratrol has variable effects on the expression of anti-oxidant molecules in both primary trophoblast and primary HUVECs.

### Resveratrol reduces pro-inflammatory cytokine expression and secretion from primary cytotrophoblasts

**Figure 4:**
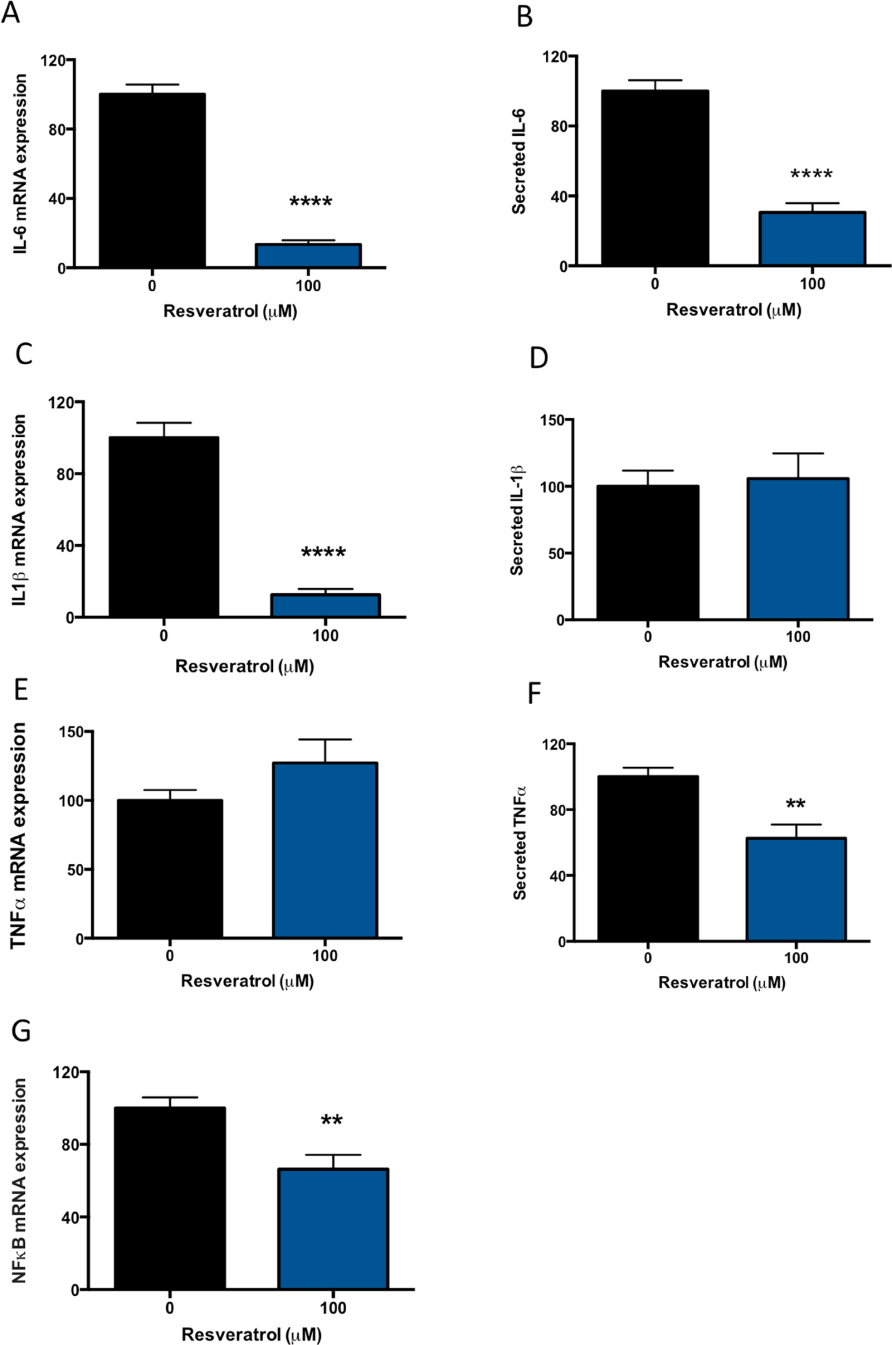
Resveratrol inhibits inflammatory molecules in primary cytotrophoblast. Preeclampsia is a condition associated with significant inflammation. Therefore we assessed the ability for resveratrol to reduce pro-inflammatory cytokine mRNA expression and secretion. In primary trophoblast, we found that resveratrol significantly reduced (**A**) IL-6 mRNA and (**B**) protein secretion. Whilst resveratrol significantly reduced (**C**) IL-1β mRNA expression, there was no significant effect on IL-1β secretion (**D**). TNFα mRNA expression was not altered by resveratrol (**E**), however secreted TNFα was significantly reduced (**F**). Finally a significant reduction in (**G)** NFκB mRNA expression was also significantly reduced. Data expressed as mean ± SEM. **p < 0.01, ****p < 0.0001 compared to 0 resveratrol (control). N = 3–5 experimental replicates, with each n taken from a separate patient isolation.

Inflammatory cytokines are found in high abundance in the circulation of patients with preeclampsia^9^. It is possible that they are placentally derived^27–29^. As such, we sought to investigate whether resveratrol would have anti-inflammatory effects in primary cytotrophoblasts. We measured the mRNA expression of inflammatory molecules in primary cytotrophoblast treated with resveratrol and found that resveratrol significantly reduced the mRNA expression of IL-6 (Fig. 4A), IL-1β (Fig. 4C), and NFκB (Fig. 4G), whilst having no significant effect on TNFα mRNA expression (Fig. 4E). We also measured the secreted levels of IL-6, IL-1β and TNFα. IL-6 (Fig. 4B) and TNFα (Fig. 4F) were significantly reduced by resveratrol treatment, whilst no change in secreted IL-1β was observed (Fig. 4D). Thus, this data demonstrates that resveratrol decreases the expression and secretion of inflammatory cytokines in primary cytotrophoblasts.

### The effects of resveratrol on endothelial dysfunction

Given that endothelial dysfunction plays an important role in preeclampsia, we examined whether the effects of resveratrol on endothelial dysfunction using several established *in vitro* models^30–33^.

**Figure 5:**
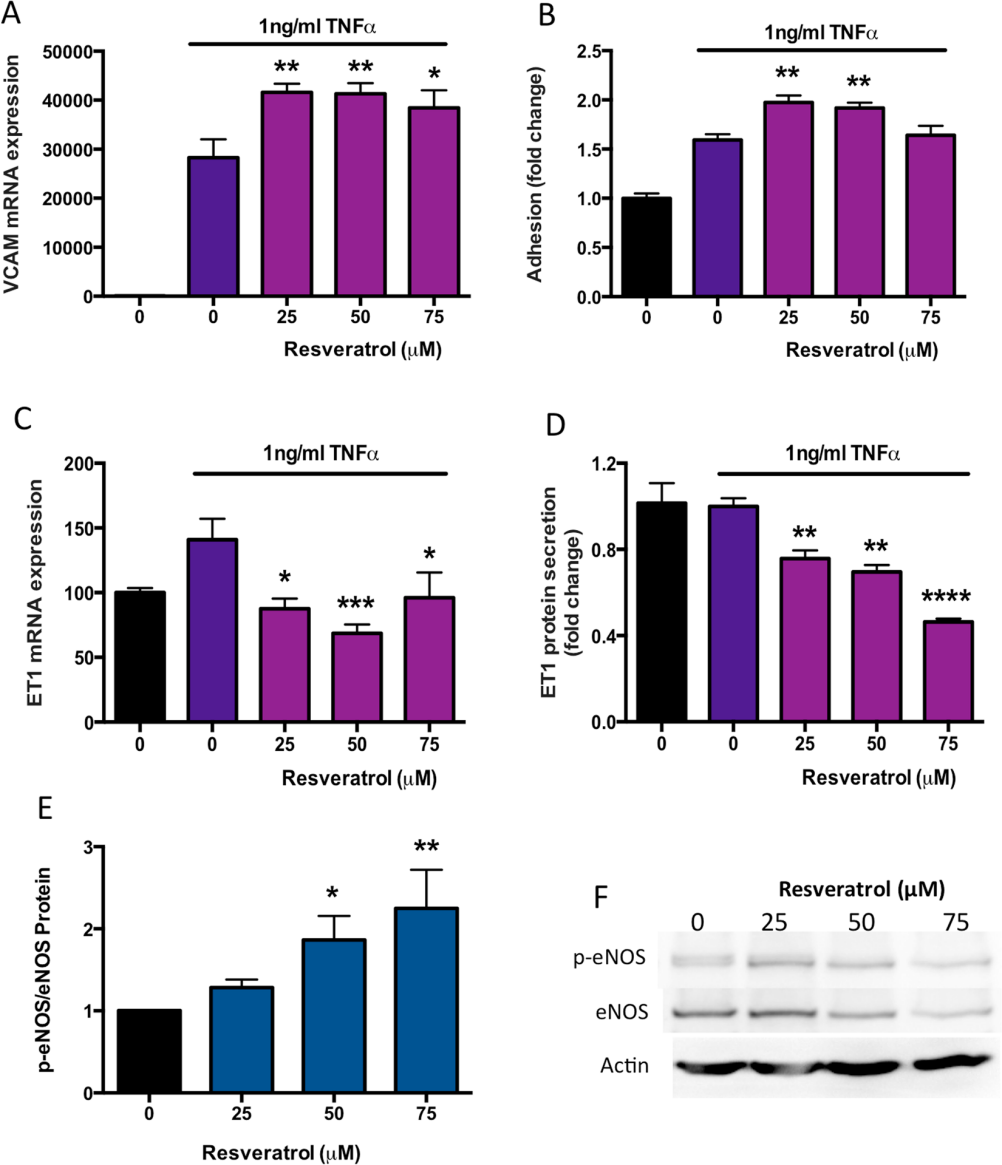
The effects of resveratrol on endothelial dysfunction. Preeclampsia is associated with significant endothelial dysfunction induced by a myriad of pro-inflammatory and anti-angiogenic molecules. In these experiments, we used TNFα to induce endothelial dysfunction *in vitro*. TNFα increased the mRNA expression of (**A**) VCAM1 300-fold beyond control, and this increase was further stimulated by resveratrol treatment. The increased expression in VCAM1 expression was reflected in the PBMC adhesion assay where (**B**) resveratrol significantly increased adhesion of PBMCs to TNFα treated HUVECs. Endothelin-1 is a potent vasoconstrictor elevated in the secretion of patients with preeclampsia. TNFα treatment increased (**C**) ET-1 mRNA expression, and this effect was returned to control levels by the addition of resveratrol. (**D**) Resveratrol also potently decreased ET-1 protein secretion from TNFα treated HUVECs. Finally we assessed the phosphorylation of eNOS (p-eNOS) to total eNOS protein levels in non-dysfunctional primary HUVECs treated with resveratrol. Western blot with densitometric analysis indicated that resveratrol significantly increased the peNOS/eNOS ratio in primary HUVECs (**E**,**F**). Data expressed as mean ± SEM. *p < 0.05, **p < 0.01, ***p < 0.001 compared to TNFa alone (0 resveratrol). (**F**) Shows a representative western blot from n = 1 HUVEC experiment, whilst E depicts densitometric analysis of n = 3 separate HUVEC western blots. As indicated in the materials and methods, western blots were cut before antibody exposure and therefore cropped blots are displayed. N = 3–5 experimental replicates, with each n taken from a separate patient isolation.

We administered TNFα to HUVECs to mimic endothelial dysfunction *in vitro*. We measured vascular cell adhesion molecule (VCAM-1), a widely accepted marker of endothelial dysfunction^34, 35^. As expected, in the presence of 1 ng/ml TNFα, VCAM-1 expression was induced 300-fold compared to cells with no TNFα (Fig. 5A) indicating significant endothelial dysfunction. Surprisingly, resveratrol modestly but significantly increased TNFα-induced VCAM-1 expression further (Fig. 5A). We next performed an adhesion assay where we repeated the same experiment but added peripheral blood mononuclear cells (PBMCs) isolated from pregnant women. In agreement with the VCAM-1 findings we found resveratrol also significantly increased PBMC adhesion to primary HUVECs in the presence of TNFα (Fig. 5B).

Endothelin-1 (ET-1) is a vasoconstrictor that is secreted from the endothelium into the circulation and has been shown to be elevated in the circulation of women with preeclampsia. Whilst TNFα did not significantly increase ET-1 mRNA expression, resveratrol significantly reduced endothelial ET-1 expression compared to TNFα alone (Fig. 5C). Significantly reduced ET-1 protein secretion was also observed in a dose-dependent manner with resveratrol treatment (Fig. 5D).

Endothelial Nitric Oxide synthase (eNOS) is the enzyme responsible for generation of nitric oxide (NO) in the endothelium. NO has a very short half-life and is a potent vasodilator. Increased phosphorylation of eNOS (p-eNOS, the active form) to total eNOS ratio *in vivo* would be expected to enhance vasodilation (preventing or reversing hypertension). We assessed whether resveratrol altered the p-eNOS/eNOS ratio in primary HUVECs. Indeed, we found a significant dose-dependent induction of p-eNOS/eNOS in the presence of resveratrol (Fig. 5E,F) and confirmed no negative impact of treatment via MTS (Supplementary Figure 1D).

In summary, we have found the effects of resveratrol in the endothelium are variable. It appeared to increase the expression of VCAM-1 and the adhesion of PBMCs to the endothelium. However, it had actions that could facilitate vasodilation, which would be beneficial.

## Discussion

Resveratrol is an anti-oxidant molecule previously proposed as a potential therapeutic for preeclampsia. Here we demonstrate for the first time that resveratrol potently reduces the predominant sFlt1 variant expressed in placenta (sFlt-1 e15a) as well as reducing soluble endoglin secretion from both primary trophoblast and primary HUVECs. In addition, we show that resveratrol has differential effects on anti-oxidant molecules in primary trophoblast and primary HUVECs, and that it significantly reduces secretion of the vasoconstrictor endothelin-1 (ET-1), and improves the p-eNOS/eNOS ratio in primary HUVECs.

sFlt-1 is the soluble form of the VEGF receptor (VEGFR1), released in low levels during normal pregnancy and significantly upregulated in preeclampsia where it binds and neutralizes pro-angiogenic molecules VEGF and PlGF in the circulation^36^. Previous data demonstrated that Resveratrol reduced sFlt-1 secretion from placental explants and primary HUVECs^16^. There are multiple splice variants of sFlt-1^22^. sFlt-1 e15a is the most abundantly expressed sFlt-1 isoform present in placenta, with greater than 95% of FLT-1 variants in placenta being the e15a isoform^22^. Importantly, we demonstrate here that as well as reducing total sFlt-1, resveratrol potently reduces sFlt-1 e15a mRNA expression and protein secretion from primary trophoblast. In addition, we show that resveratrol also significantly reduces sFlt-1 e15a and sFlt-1 i13 mRNA expression in primary HUVECs, highlighting resveratrol’s ability to reduce sFlt-1 transcripts as its likely mechanism for reducing sFlt-1 secretion.

In 2006, soluble endoglin was identified as a critical player in severe preeclampsia^6^. It is significantly up-regulated in the serum of preeclamptic women and correlates with disease severity^36^. Adenoviral over-expression of sEng in combination with sFlt1 in pregnant rats induced the full spectrum of clinical features observed in severe human preeclampsia: hypertension, proteinuria (ie renal disease), abnormal liver function, cerebra oedema, thrombocytopenia (low platelets) and fetal growth restriction^6^. Here we provide the first evidence that resveratrol can significantly reduce sEng secretion from both primary trophoblast and primary HUVECs. Our expression data suggests that this reduction occurs via its ability to either quench endoglin expression and endoglin protease MMP14 expression^17, 37^ (as seen in primary trophoblast) or reduce MMP14 expression whilst up-regulating TIMP3 (MMP inhibitor) expression (as seen in primary HUVECs). Together our data demonstrates that resveratrol reduces both sFlt-1 and sEng secretion from primary trophoblast and primary HUVECs. In the current study we did not examine whether resveratrol could reduce sFlt-1 and sEng secretion from preeclamptic placentas, which we note as a limitation of this work. However, in previous studies we have shown that the effect of drug treatments on sFlt-1 secretion is paralleled between normal healthy term samples and preeclamptic samples^38^. Therefore we expect that resveratrol would also significantly reduce secretion of sFlt-1 and sEng from preeclamptic placentas *in vitro*.

Resveratrol is widely described as an anti-oxidant^11, 12^ and therefore we assessed its ability to up-regulate anti-oxidant defenses in primary trophoblast. Interestingly, at a dose of 100 μM resveratrol, we observed no significant effect on anti-oxidant mRNA expression in primary trophoblast and a significant decline in HO-1 protein expression. This was different to our observations in primary HUVECs, where we observed significantly increased mRNA expression of NQO1, GCLC and HO-1 but no effect on protein expression following resveratrol treatment. Previous data in primary HUVECs suggests a significant increase in both HO-1 mRNA and protein at 100 μM^16^. In our hands 100 μM resveratrol induced a significant decline in HUVEC viability and therefore we were unable to match the dose used in the previous publication in our experiments. Nevertheless, our data suggests differential responses to resveratrol with regards to anti-oxidant defenses between primary trophoblast and primary HUVECs.

We also examined the effect of resveratrol on placental cytokine expression given preeclampsia is associated with significant inflammation. We found that resveratrol reduced trophoblast expression of IL-6, IL-1β and NfκB, whilst also reducing trophoblast secretion of IL-6 and TNFα. This is similar to findings in non-human primates, where administration of resveratrol to pregnant animals normalized expression of placental pro-inflammatory cytokines and chemokines compared to animals on a high fat western-style diet^39^.

Endothelial dysfunction is a key step in the pathogenesis of preeclampsia and leads to the manifestation of this disease clinically. Some data already suggests that resveratrol may have positive effects on endothelial dysfunction. In this report we used 1 ng/ml TNFα to induce endothelial dysfunction. In primary HUVECs we found that TNFα significantly increased expression of VCAM-1 and that this expression was further increased by resveratrol – an effect that was also apparent in our PBMC adhesion assay. This finding is in contrast to previously published data that suggested resveratrol reduces THP (monocyte cell line) adhesion to primary HUVECs^40^. Of note however, in that report, resveratrol was used in a preventative fashion administered 2 hours prior to the TNFα insult, whereas in our study, cells were rendered dysfunctional (to mimic the endothelial dysfunction of preeclampsia) before resveratrol was added. This may account for the difference in results.

ET-1 is a vasopressor synthesized by endothelial cells in culture and *in vivo*. Importantly, ET-1 is significantly increased in the circulation of patients with preeclampsia and thought to be a key mediator of hypertension in this disease^41, 42^. In this study we demonstrate that resveratrol significantly reduced both the mRNA and protein secretion of TNF-α^41, 42^ induced ET-1 in primary HUVECs. This finding is in accord with previous data from rats, where it was demonstrated that resveratrol significantly reduced angiotensin-induced ET-1 by targeting ERK1/2^43^ and in HUVECs where it was shown that resveratrol reduced cyclic-strain induced ET-1 gene expression^44^. In support of potential positive effects of resveratrol on hypertension, we also confirmed that resveratrol enhances phosphorylation of eNOS in primary HUVECs^45^, suggesting it may increase production of the vasodilatory molecule nitric oxide.

A concern for the possible use of resveratrol to treat disorders of pregnancy is the outcome of the Roberts *et al*.^39^ study on resveratrol supplementation in pregnant nonhuman primates. In that study, resveratrol was administered to animals consuming a 35% fat diet (western-style diet) for 3 months preceding the breeding season and continued throughout pregnancy. Whilst resveratrol improved some maternal factors (caused weight loss, improved glucose tolerance, increased uterine artery blood flow, decreased placental inflammation), it had a concerning effect on the fetus. Pancreatic mass was enlarged by 42%, with a 12-fold increase in proliferation of pancreatic cells. Although the authors could not reconcile the reason behind the increased pancreatic mass, this study raises caution around the use of resveratrol during pregnancy in humans. We would also note that while this indeed raises concerns, the number of animals in that study was modest so it is difficult to know whether this applies in humans and whether this is sufficient data to rule resveratrol out as a potential treatment for preeclampsia. Unlike that experimental design, we would envisage that to treat preeclampsia, resveratrol would only need to be given for a few weeks in the second half of pregnancy.

In conclusion, our data using primary human trophoblast and endothelial cells suggests that resveratrol offers positive effects in reducing the anti-angiogenic molecules of preeclampsia, sFlt-1 and sEng. In addition, we report, that although it has limited effects on anti-oxidant molecules in placenta and increases VCAM1 expression in endothelial cells, it dose dependently reduces ET-1 secretion and phosphorylation of eNOS. Resveratrol has potential as a treatment for preeclampsia.

## Electronic supplementary material

Supplementary material

## References

[1] Redman, C. W. & Sargent, I. L. Latest advances in understanding preeclampsia. *Science* **308**, 1592–1594, doi:308/5728/159210.1126/science.1111726 (2005). 10.1126/science.1111726 15947178

[2] Sibai, B., Dekker, G. & Kupferminc, M. Pre-eclampsia. *Lancet* **365**, 785–799, doi:S0140-6736(05)17987-2 10.1016/S0140-6736(05)17987-2 (2005). 10.1016/S0140-6736(05)17987-2 15733721

[3] Maynard S (2003). Excess placental soluble fms-like tyrosine kinase 1 (sFlt-1) may contribute to endothelial dysfunction, hypertension, and proteinuria in pre-eclampsia. The Journal of Clinical Investigation.

[4] Maynard, S. E. & Karumanchi, S. A. Angiogenic factors and preeclampsia. *Semin Nephrol* **31**, 33–46, doi:S0270-9295(10)00174-9 10.1016/j.semnephrol.2010.10.004 (2011). 10.1016/j.semnephrol.2010.10.004 PMC3063446 21266263

[5] Nagamatsu T (2004). Cytotrophoblasts up-regulate soluble fms-like tyrosine kinase-1 expression under reduced oxygen: an implication for the placental vascular development and the pathophysiology of preeclampsia. Endocrinology.

[6] Venkatesha, S. *et al*. Soluble endoglin contributes to the pathogenesis of preeclampsia. *Nat Med* **12**, 642–649, doi:nm1429 10.1038/nm1429 (2006). 10.1038/nm1429 16751767

[7] Powe, C. E., Levine, R. J. & Karumanchi, S. A. Preeclampsia, a disease of the maternal endothelium: the role of antiangiogenic factors and implications for later cardiovascular disease. *Circulation* **123**, 2856–2869, doi:123/24/2856 10.1161/CIRCULATIONAHA.109.853127 (2011). 10.1161/CIRCULATIONAHA.109.853127 PMC3148781 21690502

[8] Young BC, Levine RJ, Karumanchi SA (2010). Pathogenesis of preeclampsia. Annu Rev Pathol.

[9] Chaiworapongsa T, Chaemsaithong P, Yeo L, Romero R (2014). Pre-eclampsia part 1: current understanding of its pathophysiology. Nature reviews. Nephrology.

[10] Diaz-Gerevini GT (2016). Beneficial action of resveratrol: How and why?. Nutrition.

[11] Csiszar A (2011). Anti-inflammatory effects of resveratrol: possible role in prevention of age-related cardiovascular disease. Annals of the New York Academy of Sciences.

[12] Vang O (2011). What is new for an old molecule? Systematic review and recommendations on the use of resveratrol. PLoS One.

[13] Poudel R (2013). Effects of resveratrol in pregnancy using murine models with reduced blood supply to the uterus. PLoS One.

[14] McCarthy FP, Kingdom JC, Kenny LC, Walsh SK (2011). Animal models of preeclampsia; uses and limitations. Placenta.

[15] Zou Y (2014). Resveratrol inhibits trophoblast apoptosis through oxidative stress in preeclampsia-model rats. Molecules.

[16] Cudmore, M. J. *et al*. Resveratrol inhibits the release of soluble fms-like tyrosine kinase (sFlt-1) from human placenta. *Am J Obstet Gynecol* **206**, 253 e210–255, doi:10.1016/j.ajog.2011.11.010 (2012). 10.1016/j.ajog.2011.11.010 22197494

[17] Kaitu’u-Lino, T. J. *et al*. MMP-14 Is Expressed in Preeclamptic Placentas and Mediates Release of Soluble Endoglin. *Am J Pathol* **180**, 888–894, doi:S0002-9440(11)01077-7 10.1016/j.ajpath.2011.11.014 (2012). 10.1016/j.ajpath.2011.11.014 22296769

[18] Kaitu’u-Lino TJ (2014). Characterization of protocols for primary trophoblast purification, optimized for functional investigation of sFlt-1 and soluble endoglin. Pregnancy Hypertens.

[19] Brownfoot FC, Hannan N, Onda K, Tong S, Kaitu’u-Lino T (2014). Soluble endoglin production is upregulated by oxysterols but not quenched by pravastatin in primary placental and endothelial cells. Placenta.

[20] Onda K (2015). Sofalcone upregulates the nuclear factor (erythroid-derived 2)-like 2/heme oxygenase-1 pathway, reduces soluble fms-like tyrosine kinase-1, and quenches endothelial dysfunction: potential therapeutic for preeclampsia. Hypertension.

[21] Whitehead CL (2011). Placental expression of a novel primate-specific splice variant of sFlt-1 is upregulated in pregnancies complicated by severe early onset pre-eclampsia. BJOG: an international journal of obstetrics and gynaecology.

[22] Jebbink J (2011). Expression of placental FLT1 transcript variants relates to both gestational hypertensive disease and fetal growth. Hypertension.

[23] Palmer KR (2015). Placental-Specific sFLT-1 e15a Protein Is Increased in Preeclampsia, Antagonizes Vascular Endothelial Growth Factor Signaling, and Has Antiangiogenic Activity. Hypertension.

[24] Leinonen HM, Kansanen E, Polonen P, Heinaniemi M, Levonen AL (2014). Role of the keap1-nrf2 pathway in cancer. Advances in cancer research.

[25] Anuranjani, Bala M (2014). Concerted action of Nrf2-ARE pathway, MRN complex, HMGB1 and inflammatory cytokines - Implication in modification of radiation damage. Redox biology.

[26] Koskenkorva-Frank TS, Weiss G, Koppenol WH, Burckhardt S (2013). The complex interplay of iron metabolism, reactive oxygen species, and reactive nitrogen species: insights into the potential of various iron therapies to induce oxidative and nitrosative stress. Free radical biology & medicine.

[27] Conrad KP, Benyo DF (1997). Placental cytokines and the pathogenesis of preeclampsia. American journal of reproductive immunology.

[28] Rusterholz C, Hahn S, Holzgreve W (2007). Role of placentally produced inflammatory and regulatory cytokines in pregnancy and the etiology of preeclampsia. Semin Immunopathol.

[29] Taki A (2012). Expression of angiogenesis-related factors and inflammatory cytokines in placenta and umbilical vessels in pregnancies with preeclampsia and chorioamnionitis/funisitis. Congenital anomalies.

[30] Brownfoot FC (2015). Metformin as a prevention and treatment for preeclampsia: effects on soluble fms-like tyrosine kinase 1 and soluble endoglin secretion and endothelial dysfunction. Am J Obstet Gynecol.

[31] Brownfoot, F. C. *et al*. Effects of Pravastatin on Human Placenta, Endothelium, and Women With Severe Preeclampsia. *Hypertension* **66**, 687–697, discussion 445, doi:10.1161/HYPERTENSIONAHA.115.05445 (2015). 10.1161/HYPERTENSIONAHA.115.05445 26222708

[32] Brownfoot FC (2015). YC-1 reduces placental sFlt-1 and soluble endoglin production and decreases endothelial dysfunction: A possible therapeutic for preeclampsia. Molecular and cellular endocrinology.

[33] Onda, K. *et al*. Sofalcone Upregulates the Nuclear Factor (Erythroid-Derived 2)-Like 2/Heme Oxygenase-1 Pathway, Reduces Soluble fms-Like Tyrosine Kinase-1, and Quenches Endothelial Dysfunction: Potential Therapeutic for Preeclampsia. *Hypertension*, doi:10.1161/HYPERTENSIONAHA.114.04781 (2015). 10.1161/HYPERTENSIONAHA.114.04781 25667213

[34] Austgulen R, Lien E, Vince G, Redman CW (1997). Increased maternal plasma levels of soluble adhesion molecules (ICAM-1, VCAM-1, E-selectin) in preeclampsia. Eur J Obstet Gynecol Reprod Biol.

[35] Chaiworapongsa T (2002). Soluble adhesion molecule profile in normal pregnancy and pre-eclampsia. J Matern Fetal Neonatal Med.

[36] Levine, R. J. *et al*. Serum sFlt1 concentration during preeclampsia and mid trimester blood pressure in healthy nulliparous women. *Am J Obstet Gynecol* **194**, 1034–1041, doi:S0002-9378(05)01739-4 10.1016/j.ajog.2005.10.192 (2006). 10.1016/j.ajog.2005.10.192 16580293

[37] Hawinkels LJ (2010). Matrix metalloproteinase-14 (MT1-MMP)-mediated endoglin shedding inhibits tumor angiogenesis. Cancer Res.

[38] Onda, K. *et al*. Proton Pump Inhibitors Decrease Soluble fms-Like Tyrosine Kinase-1 and Soluble Endoglin Secretion, Decrease Hypertension, and Rescue Endothelial Dysfunction. *Hypertension*, doi:10.1161/HYPERTENSIONAHA.116.08408 (2017). 10.1161/HYPERTENSIONAHA.116.08408 28115513

[39] Roberts VH (2014). Beneficial and cautionary outcomes of resveratrol supplementation in pregnant nonhuman primates. FASEB J.

[40] Pendurthi UR, Rao LV (2002). Resveratrol suppresses agonist-induced monocyte adhesion to cultured human endothelial cells. Thrombosis research.

[41] George EM, Granger JP (2011). Endothelin: key mediator of hypertension in preeclampsia. American journal of hypertension.

[42] George EM, Palei AC, Granger JP (2012). Endothelin as a final common pathway in the pathophysiology of preeclampsia: therapeutic implications. Current opinion in nephrology and hypertension.

[43] Chao HH (2005). Resveratrol inhibits angiotensin II-induced endothelin-1 gene expression and subsequent proliferation in rat aortic smooth muscle cells. European journal of pharmacology.

[44] Liu JC, Chen JJ, Chan P, Cheng CF, Cheng TH (2003). Inhibition of cyclic strain-induced endothelin-1 gene expression by resveratrol. Hypertension.

[45] Wallerath T (2002). Resveratrol, a polyphenolic phytoalexin present in red wine, enhances expression and activity of endothelial nitric oxide synthase. Circulation.

