# Supplementary material for: Resveratrol inhibits release of soluble fms-like tyrosine kinase (sFlt-1) and soluble endoglin and improves vascular dysfunction – implications as a preeclampsia treatment
Source: Sci Rep. 2017 May 12;7:1819. doi: 10.1038/s41598-017-01993-w (PMC5431923; doi:10.1038/s41598-017-01993-w)
Supplement: Supplementary file 1 — Supplementary material [file 41598_2017_1993_MOESM1_ESM.pdf]

Resveratrol inhibits release of soluble fms-like tyrosine kinase (sFlt-1) and soluble endoglin and improves vascular dysfunction – implications as a preeclampsia treatment

Natalie J Hannan[1], Fiona C Brownfoot[1], Ping Cannon[1], Minh Deo[1], Sally Beard[1],Tuong V Nguyen[1], Kirsten R Palmer[2], Stephen Tong[1], Tu'uhevaha J Kaitu'u-Lino[1]

[1]Translational Obstetrics Group, The Department of Obstetrics and Gynaecology, Mercy Hospital for Women, University of Melbourne, 163 Studley Rd, Heidelberg 3084, Victoria, Australia

[2]Department of Obstetrics and Gynaecology, Monash University, Monash Medical Centre, 246 Clayton Rd, Clayton, 3168, Victoria, Australia

Corresponding author

Tu'uhevaha Kaitu'u-Lino

Mercy Hospital for Women

Dept of Obstetrics and Gynaecology, University of Melbourne

163 Studley Road,

Heidelberg, VIC, 3084.

Telephone: 03 8458 4355

Email: t.klino@unimelb.edu.au

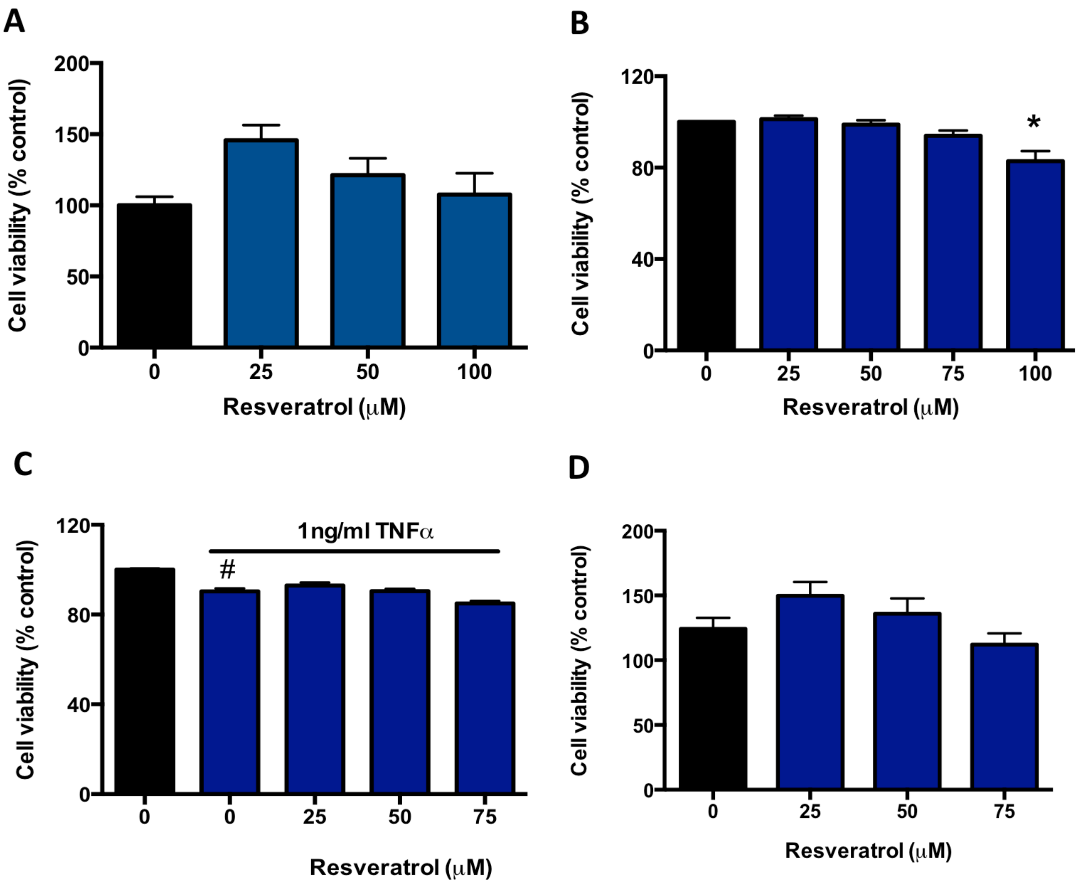

Supplementary Figure 1: Effect of resveratrol on cell viability. To determine whether cell viability was altered in the presence of resveratrol, MTS cell viability assays were undertaken for all experiments. **A)** Cell viability on primary trophoblast used for sFlt-1 and sEng secretion and mRNA expression. No significant effects of resveratrol at 25-100μM on cell viability was observed. **B)** Cell viability on primary HUVECs used for measurement of sFlt-1, sEng, IL-1β, IL-6 and TNFα secretion and mRNA expression. A minor but significant reduction in cell viability was observed in primary HUVECs at a dose of 100μM resveratrol. Therefore only doses of 25-75μM were used for experimental analysis. **C)** Cell viability of primary HUVECs used for measurement of sFlt-1 secretion and endothelial dysfunction. TNFα induced a minor but significant reduction in viability compared to control, however no additional reductions were observed in the presence of resveratrol. **D)** Cell viability of primary HUVECs used for protein analysis of HO-1 and eNOS, peNOS. No significant alterations in cell viability were observed.
